# The Permanent Relief of Tooth-ache

**Published:** 1847-02

**Authors:** 


*The Permanent Relief of Tooth-ache.* To the Editor of the British American Journal.—Sir:—In a country where so many are martyrs to this species of suffering, you will, I think, be conferring a general benefit, by making known through the medium of your journal, the following simple, and, as I have found it, successful method of securing carious teeth from the effects of cold and changeable weather, and keeping them perfectly free from pain at all times. This wonder-working remedy! consists in the daily and *habitual* use of a weak solution of creosote, saturating the tooth-brush with it and using it first; after which cold water and whatever tooth-powder the individual may be in the habit of employing.

This practice, in my own experience, and in that of others at my suggestion, I have found a very successful preventative to tooth-ache arising from the presence of carious teeth. I am rather disposed to believe, too, (contrary to the opinion of some dentists) that the carious process is suspended by its employment; but on this head I would not be confident, although Reichenbach has recorded cases of caries cured by the use of the watery solution of creosote.—"Bulletin General de Therapeutique for May, 1835." M. Fremanger is also of the same opinion as to its effects, and considers that it acts "by combining with the calcareous salts of the bones and forming a new combination, which, by its solubility, tends to disengage the areolar tissue and stop the ulceration at the proper point for the commencement of cicatrization."—"Cormack on Cresote." I wish the profession in Canada would take up the subject.—

Yours respectfully,
J. D. M'DIARMID,
Staff-Surgeon, Prescott.

B Creosote, ʒi.; Spt. Rectificat, ℥ss.; Aq. Destillat, ℥ viiss m. It may be colored with cochineal.—*Brit. Am. Jour.*

